# Frontal-to-Parietal Theta Interactions Mediate Tactile Decision-Making

**DOI:** 10.3390/life16030390

**Published:** 2026-02-28

**Authors:** Pritom Mukherjee, Sydney Apraku, Mukesh Dhamala

**Affiliations:** 1 Department of Physics and Astronomy, Georgia State University, Atlanta, GA 30303, USAsydneyapraku@gmail.com (S.A.); 2 Neuroscience Institute, Georgia State University, Atlanta, GA 30303, USA; 3 Center for Behavioral Neuroscience, Center for Diagnostics and Therapeutics, Georgia State University, Atlanta, GA 30303, USA; 4 Tri-Institutional Center for Translational Research in Neuroimaging and Data Science (TReNDS), Georgia State University, Georgia Institute of Technology, and Emory University, Atlanta, GA 30303, USA

**Keywords:** tactile stimuli, decision-making, fronto-parietal network, theta oscillations, alpha oscillations

## Abstract

Decision-making relies on coordinated neural dynamics that integrate sensory evidence with top-down control. In this EEG study, we examined sensor (scalp)-level theta and alpha-band oscillations, as well as fronto-parietal network connectivity, during a tactile spatial discrimination task. Blindfolded participants judged the lateral offset of the central dot in a three-dot array delivered to the right index finger while an EEG was recorded. Time–frequency analyses revealed that both theta and alpha power were greater for correct than incorrect decision trials during pre-stimulus and post-stimulus intervals, suggesting enhanced preparatory and mnemonic engagement during accurate decisions. Directional connectivity assessed using block (multivariate) Granger causality demonstrated significantly stronger frontal-to-parietal influence in the theta band during both pre- and post-stimulus periods for correct decisions, supporting the role of long-range theta communication for top-down control in guiding tactile judgment. These findings highlight theta-band fronto-parietal communication as a key mechanism supporting successful tactile decision-making.

## 1. Introduction

Decision-making is a cognitive process that integrates prior knowledge, sensory evidence, and expected value to produce a definitive choice aimed at accomplishing specific objectives [1]. Decision-making is an integral part of our daily lives, and the brain regions involved in this process have therefore become a key focus of interest in cognitive neuroscience [2,3,4]. Decision-making processes have long been studied by psychologists and neuroscientists, both in animals and humans. With the advancement of neuroimaging tools, researchers are leaning more towards understanding the neural underpinnings and sub-processes involved in such behavioral processes [5,6,7]. Decision-making processes have been extensively studied using visual stimuli [8,9] and auditory stimuli [10,11,12]; however, we implemented a tactile stimulus, which has been explored much less. Understanding the neural dynamics underlying tactile decisions might help us comprehend somatosensory processing, sensory-motor integration, and cognitive control that translate sensory inputs into behavioral responses.

Electroencephalography (EEG) provides a noninvasive and cost-effective approach with excellent temporal resolution, making it well-suited for investigating neural dynamics. It has been widely used to study decision-making processes [13,14] in various aspects. We are most interested in examining sensor-level activity, which contains rich neural information. A substantial body of research has established that neural oscillations in specific frequency bands play critical roles in cognitive control and decision-making processes [15,16,17,18]. Theta-band (4–7 Hz) oscillations have been implicated in top-down preparation, cognitive control, and decision-making [19,20]. Alpha-band oscillations (8–12 Hz) constitute the dominant rhythm in the human brain. They have been extensively linked to attention, information processing, and working memory maintenance [21,22,23]. Recent studies have also associated alpha oscillations with decision-making [24,25,26]. Building on these findings, the present study investigates the timing, spatial distribution, and influence of theta and alpha oscillations during tactile perceptual decision-making. We hypothesize that both theta and alpha power will mediate successful decision-making, reflecting their roles in cognitive control and attentional engagement during successful task performance.

Neuroimaging studies have also revealed the presence of large-scale, organized brain networks that play a critical role in supporting a wide range of functions [27,28,29]. The involvement of the fronto-parietal network in visuospatial working memory has been consistently demonstrated across multiple neuroimaging studies [30,31,32]. In addition, a study examining complex value-based decision-making reported notable activations within this network [33]. Evidence from a meta-analysis of functional magnetic resonance imaging studies further supports the role of the fronto-parietal network in perceptual decision-making [34]. Based on these findings, we hypothesize that fronto-parietal network interactions will play a key role in tactile decision-making and mediate decision accuracy.

## 2. Materials and Methods

### 2.1. Participants

The experiment involved 15 healthy right-handed participants (12 males, 3 females). Their ages were in the range of 18 to 42 years (mean = 24.7 years, standard deviation: 5.7 years). Two subjects had to be excluded from the final analysis due to excessive noise and artifacts in their data. For EEG spectral power and directional connectivity analyses, this sample size is within the range commonly reported in the literature. Moreover, we employed a linear mixed-effects model for statistical analysis (see Section 2.9), which accounts for both inter-subject and intra-subject variability. Informed consent was collected from the participants, and the original study [5] was approved by the Institutional Review Boards of Emory University and Georgia State University. The current study is the reanalysis of these deidentified EEG data recordings.

### 2.2. Tactile Stimulation

**Figure 1:**
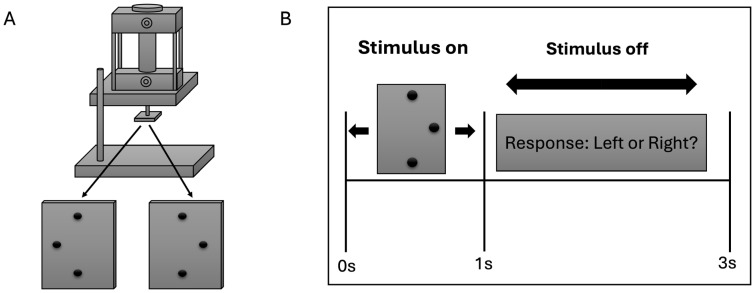
Stimuli and Task Paradigm. (**A**) Stimuli: an MRI/EEG-compatible pneumatic stimulator. The stimuli were mounted face down on the square stage at the bottom of the drive shaft. The finger mold used to immobilize the finger was mounted to the device’s base. (**B**) Task Paradigm: the central dot of a raised three-dot array was displaced either leftward or rightward. Tactile stimuli were delivered to the right index fingerpad using a pneumatically actuated stimulator for a duration of 1 s (on interval), after which participants provided their responses during a subsequent 2 s period (off interval).

The experimental configuration closely followed that used in a previous fMRI study [35], with modifications appropriate for EEG acquisition. Tactile stimulation was delivered to the right index fingerpad using a pneumatically driven stimulator (Figure 1A), with the long axis of the stimulus array aligned along the finger. The stimulus consisted of a raised three-dot array mounted on a square base plate measuring 20 mm × 20 mm. The two outer dots were separated by 4 mm, while the central dot was offset by 1.94 mm to either the left or right of the line connecting the outer dots. All dots protruded 0.64 mm above the surface of the plate. During stimulation, the participant’s right index finger was immobilized in a supine (palmar-up) orientation using a finger mold mounted on the base of the stimulator. Thick, double-sided adhesive tape was used to secure the finger in place and served as padding to ensure comfort. A rotating disk mounted on the stimulator allowed the stimulus plate to be rotated by 180°, enabling rapid switching between leftward and rightward offsets. Care was taken to ensure that the stimulus array was precisely centered on the base plate so that rotation resulted in symmetric positioning of the two stimulus configurations. Each tactile stimulus was applied to the fingerpad for 1 s. After stimulus offset, participants had 2 s to respond with a left- or right-mouse click with their left hand to indicate whether the central dot was offset to the left or right (Figure 1B). Stimulation timing and sequence were controlled using a computer program written in Presentation (Neurobehavioral Systems, Albany, CA, USA), which also provided accurate timing records for synchronization with EEG data acquisition. Throughout the experiment, participants were blindfolded and did not visually observe the stimuli at any point during the study.

### 2.3. Data Acquisition and Preprocessing

Before EEG acquisition, participants completed a practice session designed to familiarize them with the task. Practice trials were organized into blocks of 20, with leftward and rightward offsets occurring with equal probability. The practice session consisted of two such blocks. After the EEG setup, participants received a brief explanation of the basic principles of EEG recording and were instructed on how to minimize the introduction of artifacts into the ongoing EEG signals. Continuous EEG data were acquired using a Neuroscan recording system equipped with a 68-channel electrode cap, sintered AgCl electrodes, and SynAmps2 amplifiers, sampled at 1000 Hz per channel (Neuroscan Systems, Charlotte, NC, USA). Signals were digitized with a 24-bit analog-to-digital resolution. The electrode cap was positioned according to standard cranial landmarks, and recording electrodes were referenced to the right mastoid. Electrode impedances were maintained below 10 kΩ. Data were collected across 30 blocks for 7 participants, 20 blocks for 5 participants, and 10 blocks for 3 participants. Each run consisted of 20 trials with equal probability of left and right stimuli. Participants completing 30 blocks performed one run per block, whereas those completing 20 and 10 blocks performed three and six runs per block, respectively. The block structure was determined during a practice session based on participants’ self-reported fatigue tolerance, as some participants were unable to complete more than one run at a time, while others were able to complete multiple runs within a single block. All participants completed the same total number of trials. EEG data were preprocessed as follows: EEG signals were band-pass filtered between 1 and 100 Hz and notch filtered to eliminate 60 Hz line noise. Data from malfunctioning electrodes were excluded and, when appropriate, replaced through spatial interpolation using signals from neighboring electrodes.

### 2.4. Data Analysis

The analysis of the preprocessed data consisted of the following steps: (i) computation of ERPs, (ii) visualization of oscillatory dynamics, (iii) time-frequency analysis of oscillatory power, (iv) frequency band power extraction, (v) statistical analysis of oscillatory power using a linear mixed-effects model, and (vi) computation of multivariate Granger causality based on the nonparametric [36] wavelet-based spectral method. Details are provided below.

### 2.5. Event-Related Potentials (ERPs)

To examine stimulus-locked neural responses, we computed event-related potentials (ERPs) by averaging EEG data across trials and subjects. For each subject, trials within each experimental condition (Correct, Incorrect, Left, Right) were concatenated and averaged to obtain single-subject ERPs, which were then averaged across all 13 subjects to produce grand-average waveforms. The Correct condition combined Left and Right correct trials, enabling comparison of both performance accuracy (Correct vs. Incorrect) and cue laterality (Left vs. Right).

### 2.6. Spatiotemporal Visualization of Oscillatory Dynamics

To visualize the spatiotemporal evolution of alpha and theta power across the scalp, we created time-frequency heatmaps for each experimental condition. Power values were averaged across all subjects and displayed as channel-by-time matrices spanning from −500 ms to 1000 ms. Each heatmap represented one condition, with color intensity indicating the magnitude of z-scored power. These visualizations allowed us to identify when and where condition-specific differences in oscillatory activity emerged across the electrode array, revealing both the temporal dynamics and spatial topography of frequency-band-specific neural responses to the experimental manipulations. We also created topographic scalp maps to better visualize the spatial distribution across all 68 electrode locations for each experimental condition and time window.

### 2.7. Time Frequency Analysis of Oscillatory Power

We performed time-frequency decomposition of the EEG data using continuous wavelet transform (CWT) with complex Morlet wavelets. The analysis computed spectral power across frequencies from 1 to 50 Hz for each trial, electrode, and experimental condition, following established wavelet analysis methods [37]. Classical baseline normalization was used, which assumes that stimulus-induced brain activity adds linearly to ongoing baseline activity [38]. For each frequency, we computed the mean and standard deviation of power during the pre-stimulus period, then subtracted the baseline mean from all time points and divided by the baseline standard deviation. This approach expresses stimulus-related power changes in units of standard deviations relative to baseline, enabling direct comparison of oscillatory responses across frequency bands [39].

### 2.8. Frequency Band Power Extraction

To quantify oscillatory activity in specific frequency bands, we extracted theta (4–7 Hz) and alpha (8–12 Hz) power from the time-frequency decompositions for each subject and experimental condition. We computed band-specific power separately for two temporal windows of interest: a pre-stimulus baseline period (−500 ms to 0 ms) and a post-stimulus period (0 ms to 500 ms). These time-averaged power values were extracted for each condition (correct vs. incorrect decisions; left vs. right cue presentations) and visualized using violin plots after applying a signed-log transformation to display the distribution of power values across subjects and channels. All statistical analyses were performed on the original z-scored data.

### 2.9. Statistical Analysis Using Linear Mixed-Effects Models

To test for statistically significant differences in theta and alpha power between experimental conditions, we employed a linear mixed-effects (LME) model. The model included Condition (e.g., Correct vs. Incorrect) as a fixed effect, with random intercepts for both Subject and Channel to account for baseline differences in power across individuals and electrode locations. This approach controls for non-independence in the data arising from repeated measures within subjects and across channels [40]. The statistical significance of condition effects was evaluated using the *p*-values associated with the fixed-effect coefficients, with α = 0.05 as the significance threshold. This modeling framework allowed us to make valid statistical inferences while appropriately accounting for the nested structure of EEG data.

### 2.10. Block Granger Causality and Coherence Analysis

To understand directional information flow across frontal and parietal regions for different conditions and neural oscillations, we performed block Granger causality [41,42,43] analysis on EEG signals from frontal (channels 11, 12, 13) and parietal (channels 50, 51, 52) electrode clusters.

Block Granger causality in the Geweke-formulation was employed since it appropriately mitigates major volume conduction–related confounds by isolating conditionally independent, time-delayed predictive influences. Volume conduction refers to the instantaneous spread of electrical activity through brain tissue and the scalp, causing multiple EEG sensors to record mixtures of the same underlying neural sources. This spatial mixing can lead to spurious correlations and misleading connectivity estimates that do not reflect true physiological interactions. Block Granger causality addresses this issue by incorporating all signals into a multivariate framework that conditions causal influences on other observed sources, thereby reducing the impact of shared or common signals [44,45]. By explicitly relying on time-lagged prediction, the method also theoretically discounts near-instantaneous, zero-lag correlations characteristic of spatial mixing. Spectral Granger causality measures the directional influence from one region to another [46,47], whereas block coherence between regions reflects frequency-specific inter-regional synchrony among oscillatory neuronal processes. We used the nonparametric method, which avoids potential issues of model misspecification. We analyzed connectivity patterns separately for the pre-stimulus baseline (−500 to 0 ms) and post-stimulus response (0 to 500 ms) time windows, focusing on the theta (4–7 Hz) and alpha (8–12 Hz) frequency bands, which have been implicated in cognitive processing and decision-making. These analyses were performed independently for each experimental condition (Correct decisions, Incorrect decisions, Left cues, Right cues) to examine how long-range communication patterns differ across behavioral outcomes and stimulus types. This block-based approach captures coordinated activity across multiple channels within each region, providing a theoretically elegant estimate of long-range neural activity.

## 3. Results

### 3.1. Group-Level Average Event-Related Potentials (ERPs)

The grand-averaged ERP waveforms at a single electrode (FCz) for correct versus incorrect trials and for left versus right responses are representative of scalp-wide patterns, with similar condition-dependent modulations (Supplementary Figure S1A,B). Both comparisons exhibit a similar overall waveform morphology, with clear stimulus-locked components following stimulus onset, and maximum activations around 200 ms. In the correct–incorrect comparison, differences in waveform amplitude are evident in the post-stimulus interval, with the two conditions showing distinct temporal profiles. In contrast, the left–right comparison reveals largely overlapping ERP waveforms across the analyzed time window, with no pronounced separation in amplitude or timing. Grand-average ERPs were also plotted for frontal and parietal regions (Supplementary Figure S7). Although the overall waveform morphology is similar to that observed at a single electrode, notable regional differences are apparent. Parietal activity appears weaker in amplitude compared to frontal activity across conditions. Additionally, in the Incorrect condition, baseline activity in the frontal region is elevated relative to the Correct condition.

### 3.2. Time-Frequency Analysis

The time–frequency representation of EEG activity at a single electrode (FCz) illustrates how spectral power evolves over time relative to stimulus onset (Supplementary Figure S1C). Increased power is primarily observed at lower frequencies (4–12 Hz) following stimulus presentation, while higher frequencies show comparatively weaker modulation across the time window.

### 3.3. Spatiotemporal Distribution of Oscillatory Power

**Figure 2:**
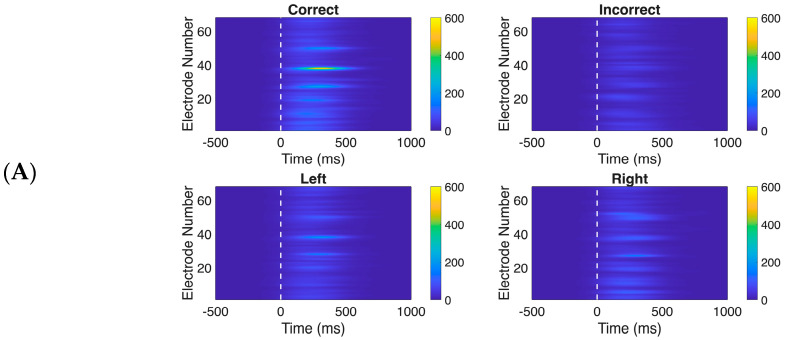

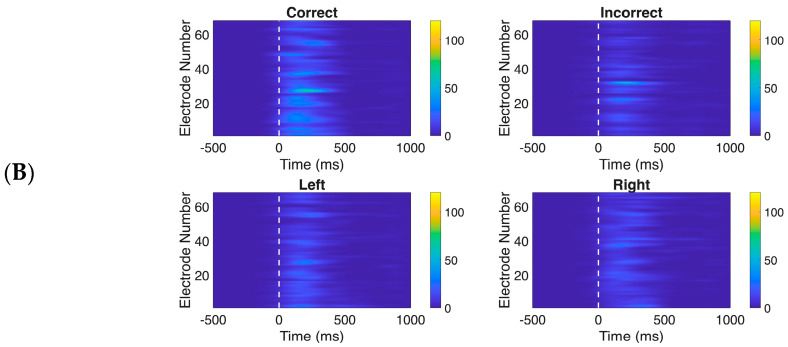
Baseline normalized oscillatory power of ERPs. (**A**) Theta band (4–7 Hz) power for all the 4 conditions: Correct, Incorrect, Left correct, Right correct; across all electrodes (1–68); (**B**) Alpha band (8–12 Hz) power for all the 4 conditions: Correct, Incorrect, Left correct, Right correct; across all electrodes (1–68).

Time-frequency analysis revealed distinct spatiotemporal patterns for both baseline-corrected theta-band (Figure 2A) and baseline-corrected alpha-band (Figure 2B) power across experimental conditions in the post-stimulus period.

The Correct condition exhibited the strongest and most spatially widespread theta power, with peak activity concentrated around electrodes 38–42 immediately following stimulus onset, while the Incorrect condition showed comparatively weaker activation. Theta power distributions across all electrodes in the left-correct and right-correct conditions did not differ much.

**Figure 3:**
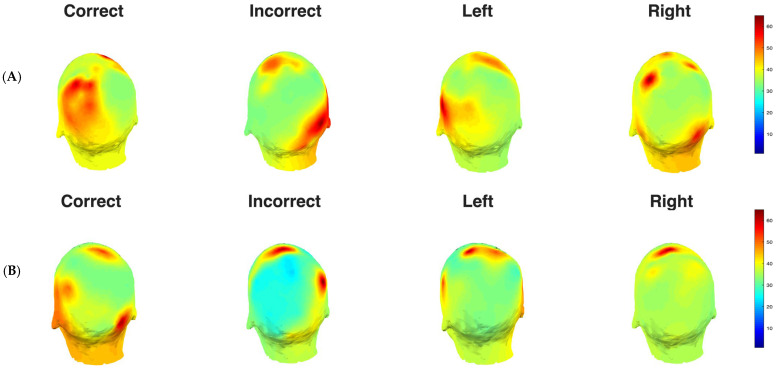
(**A**) Topographic Distribution of Theta Power. Scalp topographies showing normalized theta-band power for the post-stimulus (0 ms to 500 ms) time window across experimental conditions. (**B**) Topographic Distribution of Alpha Power. Scalp topographies showing normalized alpha-band power for the post-stimulus (0 ms to 500 ms) time window across experimental conditions.

Similarly, in the alpha band, the Correct condition exhibited the strongest alpha power, particularly around electrodes 25–35 during the 200 ms to 500 ms window, while the Incorrect condition showed relatively weaker and more spatially restricted activation. Left and Right cue conditions displayed comparable alpha patterns. We also created topographic scalp maps (Figure 3) to better visualize the spatial distribution of theta and alpha power across all 68 electrode locations for each experimental condition and time window.

### 3.4. Baseline Spatiotemporal Power Distribution

We analyzed the raw power for the ERPs, which revealed distinct baseline spatiotemporal patterns for theta and alpha oscillations across all experimental conditions (Supplementary Figure S2). This approach is necessary because baseline normalization is performed using the pre-stimulus window; thus, z-scored power did not reveal any spatiotemporal dynamics in the pre-stimulus timeframe. In this case, theta-band activity (4–7 Hz) showed a more widespread baseline distribution across electrodes. Both alpha and theta power were elevated in the left correct and right-correct conditions. For alpha-band activity (8–12 Hz), strong baseline power was evident in electrodes 60–68 (occipitoparietal regions) across all conditions, with Left and Right cue conditions showing particularly prominent activity during the pre-stimulus period (−500 ms to 0 ms). Topographic scalp maps (Supplementary Figure S3) were created for better visualization.

### 3.5. Statistical Analysis of Frequency-Band Power

**Figure 4:**
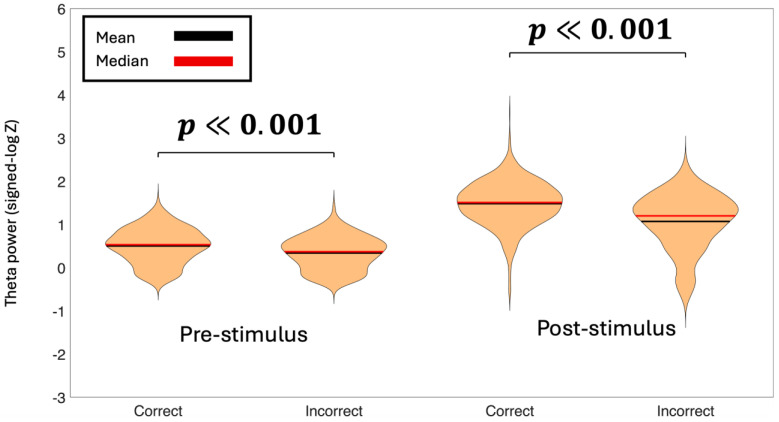
Baseline normalized average theta power during the pre-stimulus time-period (−500 ms to 0 ms) and post-stimulus time-period (0 ms to 500 ms) for correct decisions versus incorrect decisions. The *p*-values represent the significance between condition pairs. Theta power for Correct decisions is higher in both time-periods than Incorrect decisions.

**Figure 5:**
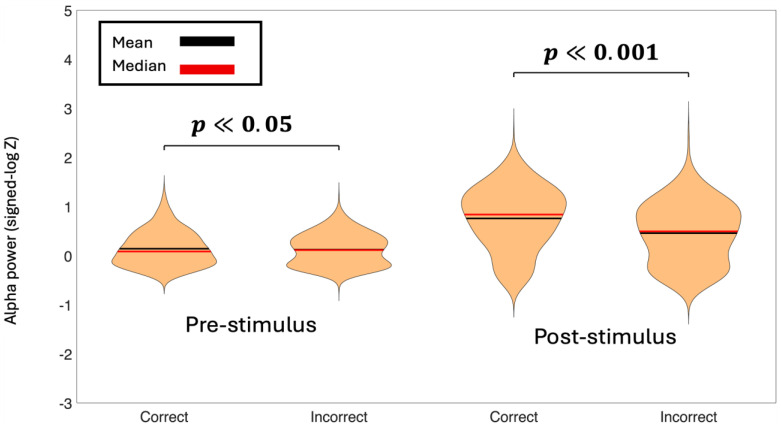
Baseline normalized average alpha power during the pre-stimulus time-period (−500 ms to 0 ms) and post-stimulus time-period (0 ms to 500 ms) for correct decisions versus incorrect decisions. The *p*-values represent the significance between condition pairs. Alpha power for Correct decisions is higher in both time-periods than Incorrect decisions.

To assess differences in theta and alpha power between experimental conditions, we employed linear mixed-effects (LME) models with spectral power as the dependent variable and Condition as a fixed effect. Random intercepts for both Subject and Channel were included to account for baseline differences in power across individuals and electrode locations. We tested comparisons between Correct vs. Incorrect decisions for the theta-band (Figure 4) and the alpha-band (Figure 5). We also tested Left vs. Right cue presentations for theta band (Supplementary Figure S4) and alpha band (Supplementary Figure S5). Statistical significance was evaluated at α = 0.05. This approach allowed us to test condition effects appropriately, since observations from the same subject and the same channel are not independent. Linear mixed-effects models revealed significant differences in oscillatory power between Correct and Incorrect decisions for both frequency bands and time windows.

Pre-stimulus theta power was significantly lower for Incorrect trials compared to Correct trials (β = −1.66, 95% CI [−2.02, −1.30], *p* < 0.001). In contrast, no significant difference in pre-stimulus theta power was observed between Left and Right correct trials (β = 0.03, 95% CI [−0.28, 0.34], *p* = 0.842). A similar pattern was observed in the alpha band during the pre-stimulus window: alpha power was significantly lower for Incorrect compared to Correct trials (β = −0.27, 95% CI [−0.45, −0.08], *p* < 0.05), whereas no significant difference was found between Left and Right correct conditions (β = −0.08, 95% CI [−0.24, 0.07], *p* = 0.279).

Post-stimulus analyses revealed stronger condition-related effects. Theta power was significantly reduced in the Incorrect condition relative to the Correct condition (β = −33.28, 95% CI [−43.43, −23.13], *p* < 0.001), while no difference was found between Left and Right correct trials (β = −0.31, 95% CI [−5.93, 5.31], *p* = 0.914). Likewise, post-stimulus alpha power was significantly lower for Incorrect compared to Correct trials (β = −6.21, 95% CI [−7.99, −4.43], *p* < 0.001), with no significant effect of cue laterality (β = −0.12, 95% CI [−1.43, 1.20], *p* = 0.861). Together, these results indicate that both pre- and post-stimulus oscillatory power in the theta and alpha bands were modulated by response accuracy rather than cue laterality.

These findings suggest that enhanced theta and alpha power, both before and after stimulus onset, are associated with successful decision-making performance.

### 3.6. Analysis of Fronto-Parietal Network Activity

**Figure 6:**
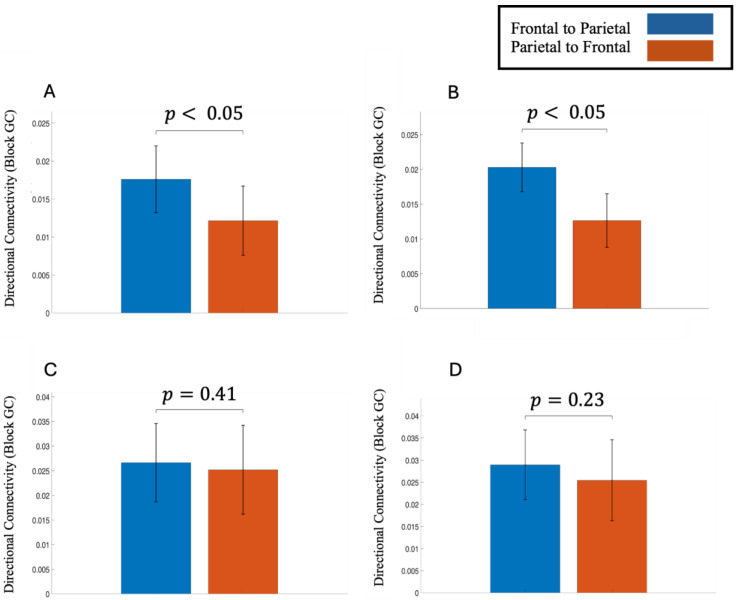
Directional information flow between frontal and parietal cortical regions for the theta band. (**A**) Correct decisions pre-stimulus period, (**B**) Correct decisions post-stimulus period, (**C**) Incorrect decisions pre-stimulus period, (**D**) Incorrect decisions post-stimulus period.

Block Granger causality analysis revealed systematic directional information flow between frontal and parietal cortical regions. In the theta band (4–7 Hz), directional flow was consistently stronger in the frontal -to-parietal direction compared with the parietal-to-frontal direction (Figure 6). This pattern was observed both before stimulus onset and during the post-stimulus decision period and was statistically significant for correct trials. A similar but weaker frontally driven pattern was also present for incorrect trials in both time windows, although these differences did not reach statistical significance. In contrast, alpha-band (8–12 Hz) directional Granger causality showed the opposite trend: parietal-to-frontal influence was greater than frontal-to-parietal influence during both pre-stimulus and post-stimulus intervals for correct and incorrect decisions (Supplementary Figure S6). However, these alpha-band directional differences were not statistically significant.

## 4. Discussion

We used a tactile discrimination task, which provided us with a unique opportunity to study neural oscillations and fronto-parietal network activity during the tactile decision-making process. EEG data were analyzed using MATLAB (version 25.1, R2025a)-based wavelet spectral methods. Event-related potentials were first computed from –500 to 1000 ms, with 0 ms marking stimulus onset, and showed clear peaks around 200 ms. Time–frequency plots were then generated for each channel and condition, revealing maximum activity in the lower frequency bands. Neural activity was averaged across electrodes to quantify mean power per condition and to identify regions showing maximal engagement during tactile discrimination. Theta and alpha power were assessed using violin plots and a linear mixed-effects model (LME). Comparisons included left-correct vs. right-correct trials and all-correct (a combination of left-correct and right-correct trials) vs. all-incorrect trials for both pre-stimulus and post-stimulus windows.

Pre-stimulus theta power and alpha power were significantly higher for correct trials compared to incorrect trials. The functional role of alpha oscillations can vary depending on the nature of the task and the stimulus context. For example, it is well-established that alpha power tends to increase when a subject’s eyes are closed rather than open [48], and in our study, this would make sense since the participants were blindfolded. Research has also shown that alpha power can increase when subjects are not engaged in a task, which led to alpha oscillations being termed as the “idling” process of brain areas [49]. Other studies argue that alpha activity helps control information flow in the brain: higher alpha power is thought to suppress activity in areas that are not needed for the task, while lower alpha power reflects active processing [50,51,52]. However, some prior studies have found that pre-stimulus alpha activity reflects a top-down preparatory state that shapes subsequent task performance, and they also provide evidence for pre-stimulus theta band activity having similar functionality [53,54,55,56,57,58]. Pre-stimulus theta power has also been linked to increased memory encoding [59]. A study that used the McGurk stimulus to understand the role of the pre-stimulus aperiodic component combined with periodic power in shaping perceptual responses found that frontal periodic theta may be linked to enhanced cognitive control or a shift in sensory precision [60]. Based on our findings, we interpret the increased activity during correct trials in the theta and alpha bands as enhanced readiness leading to more accurate tactile judgments. Time-frequency analysis clearly revealed alpha as the most dominant oscillation, which was expected, but the significant difference between correct and incorrect trials indicates a functional role for alpha oscillations during the decision-making process.

Post-stimulus theta power and alpha power were also higher for correct compared to incorrect trials. Previous studies have reported that alpha power during the working-memory retention period increases as working-memory load increases [61,62,63]. Because maintaining items in WM can support successful long-term memory formation, this suggests that alpha power during WM maintenance may be higher for items that are later remembered than for items that are later forgotten. Likewise, several studies have shown that theta power also rises with increasing WM load [64,65], and theta activity during encoding has been linked to later memory performance [66,67,68,69]. In our task, the elevated post-stimulus theta power and alpha power may reflect greater effort to build a haptic memory of the stimulus, which in turn supports more accurate left/right decisions. Both theta power and alpha power for left-correct and right-correct conditions did not differ significantly for any of the time windows.

We examined fronto-parietal network activity during correct and incorrect decisions, focusing on directional connectivity between frontal and parietal regions in the theta and alpha frequency bands. Previous studies have found that long-distance neuronal communication between brain regions during top-down control tasks, such as preparatory attention and cognitive control, is mediated by theta-band activity [70,71,72]. Moreover, an fMRI study using a willed attention paradigm found that attentional focus decisions made in frontal regions need to be relayed to parietal control areas to be carried out [73]. In our study, information flow was significantly stronger in the frontal-to-parietal direction compared with the parietal-to-frontal direction in both the pre-stimulus and post-stimulus intervals for correct decisions in the theta band. In line with previous studies, this stronger frontal-to-parietal interaction in the theta band indicates better long-range communication between the regions, which led to correct choices. Frontal-to-parietal interaction was slightly higher than parietal-to-frontal interactions for incorrect decisions as well, but the difference was not statistically significant. Thus, long-range theta activity in the fronto-parietal network seems to be a mediator of successful tactile decision-making.

In the alpha band, we noticed the opposite pattern, where parietal-to-frontal activity seemed to be marginally stronger than frontal-to-parietal activity, although this difference was not statistically significant for either condition or time window. Long-range alpha activity in the parietal-to-frontal direction did not play a crucial role in successful decision-making choices.

## 5. Conclusions

This study demonstrates that theta and alpha oscillations play critical roles in tactile perceptual decision-making. Elevated pre-stimulus theta and alpha power for correct trials suggests enhanced preparatory attentional states, while increased post-stimulus theta and alpha activity likely reflects working memory processes supporting accurate judgments. Crucially, we identified stronger frontal-to-parietal theta connectivity during correct decisions, indicating that top-down cognitive control from frontal regions to parietal areas mediates successful tactile discrimination. A key strength of this study is the integration of spectral power analysis with directional connectivity measures, providing insight into both local oscillatory dynamics and large-scale network communication during tactile decision-making. However, the relatively small sample size and use of a single tactile discrimination task may limit generalizability. Future research should examine whether these neural signatures extend to more complex tactile decisions involving multiple stimulus dimensions, explore sensor-level beta and gamma band contributions to somatosensory decision processes, and investigate how individual differences in tactile sensitivity relate to oscillatory patterns and network dynamics.

## Data Availability

Data will be made available upon reasonable request.

## Supplementary Materials

The following supporting information can be downloaded at: https://www.mdpi.com/article/10.3390/life16030390/s1, Figure S1: Grand-averaged ERPs for an electrode, FCz. (A) correct (green) and incorrect (red) decision ERPs, (B) left correct (blue) and right correct (pink) decision trial ERPs. (C) *Time–frequency representation of EEG activity at a single electrode FCz.* Time–frequency map showing changes in spectral power for correct decisions as a function of time (ms) and frequency (Hz), time-locked to stimulus onset (0 ms), Figure S2: Raw oscillatory power of ERPs. (A) Theta band (4–7 Hz) power for all the 4 conditions: Correct, Incorrect, Left correct, Right correct; (B) Alpha band (8–12 Hz) power for all the 4 conditions: Correct, Incorrect, Left correct, Right correct; across all the electrodes (1–68), Figure S3: (A) Topographic Distribution of Raw Theta Power. Scalp topographies showing normalized theta band power for pre-stimulus (−500 ms to 0 ms) time window across experimental conditions. (B) Topographic Distribution of Raw Alpha Power. Scalp topographies showing normalized alpha band power for pre-stimulus (−500 ms to 0 ms) time window across experimental conditions, Figure S4: Baseline normalized average theta power of the pre-stimulus time-period (−500 ms to 0 ms) and post-stimulus time-period (0 ms to 500 ms) for left correct decisions vs. right correct decision. The *p*-values represent the significance between condition pairs. The conditions are not significantly different from each other, Figure S5: Baseline normalized average alpha power of the pre-stimulus time-period (−500 ms to 0 ms) and post-stimulus time-period (0 ms to 500 ms) for left correct decisions vs. right correct decisions. The *p*-values represent the significance between condition pairs. The conditions are not significantly different from each other, Figure S6: Directional information flow between frontal and parietal cortical regions for the alpha band. (A) Correct decisions pre-stimulus period, (B) Correct decisions post-stimulus period, (C) Incorrect decisions pre-stimulus period, (D) Incorrect decisions post-stimulus period. Figure S7: Grand-average event-related potentials (ERPs) across conditions and regions of interest. Grand-average ERPs are shown for the (A) Left, (B) Right, (C) Correct, and (D) Incorrect conditions. For each panel, signals were averaged across frontal electrodes (11–13; blue line) and parietal electrodes (51–53; orange line). Time is shown relative to stimulus onset.
