# Supplementary material for: Frontal-to-Parietal Theta Interactions Mediate Tactile Decision-Making
Source: Life (Basel). 2026 Feb 28;16(3):390. doi: 10.3390/life16030390 (PMC13028363; doi:10.3390/life16030390)
Supplement: Supplementary file 1 [file life-16-00390-s001.zip › life-4098857-supplementary.pdf]

Supplementary Materials for "Frontal-to-Parietal Theta Interactions Mediate Tactile Decision-Making"

Pritom Mukherjee[1], Sydney Apraku[1] and Mukesh Dhamala [1,2,3,4,*]

[1]Department of Physics and Astronomy, Georgia State University, Atlanta GA USA 30303, USA
[2]Neuroscience Institute, Georgia State University, Atlanta GA USA 30303, USA
[3]Center for Behavioral Neuroscience, Center for Diagnostics and Therapeutics, Georgia State University, Atlanta, GA 30303, USA
[4]Tri-Institutional Center for Translational Research in Neuroimaging and Data Science (TReNDS), Georgia State University, Georgia Institute of Technology, and Emory University, Atlanta, GA 30303, USA
*Corresponding author: Mukesh Dhamala, Email: mdhamala@gsu.edu; Tel.: +1(404) 413-6073

The following figures provide additional visualizations supporting the results presented in the main manuscript.

1. Grand-averaged ERPs and time-frequency representation of EEG activity at a single electrode.

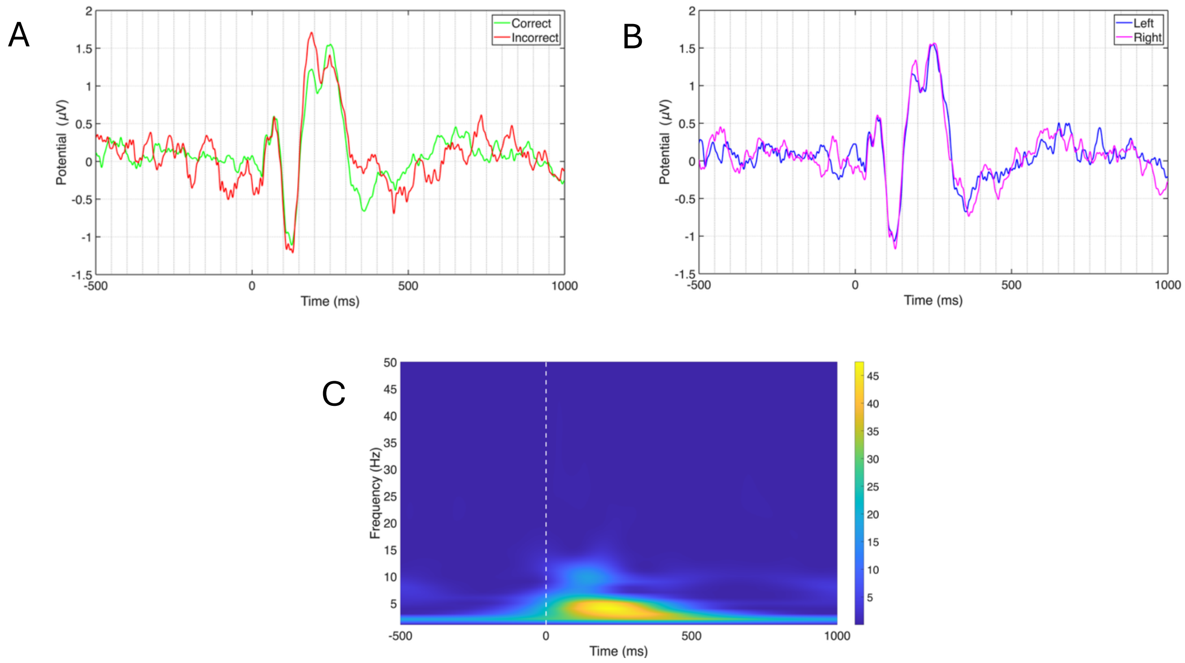

Figure S1: Grand-averaged ERPs for an electrode, FCz. (A) correct (green) and incorrect (red) decision ERPs, (B) left correct (blue) and right correct (pink) decision trial ERPs. (C) Time–frequency representation of EEG activity at a single electrode FCz. Time–frequency map showing changes in spectral power for correct decisions as a function of time (ms) and frequency (Hz), time-locked to stimulus onset (0 ms).

2. Raw oscillatory power of ERPs across the total time window for all electrodes.

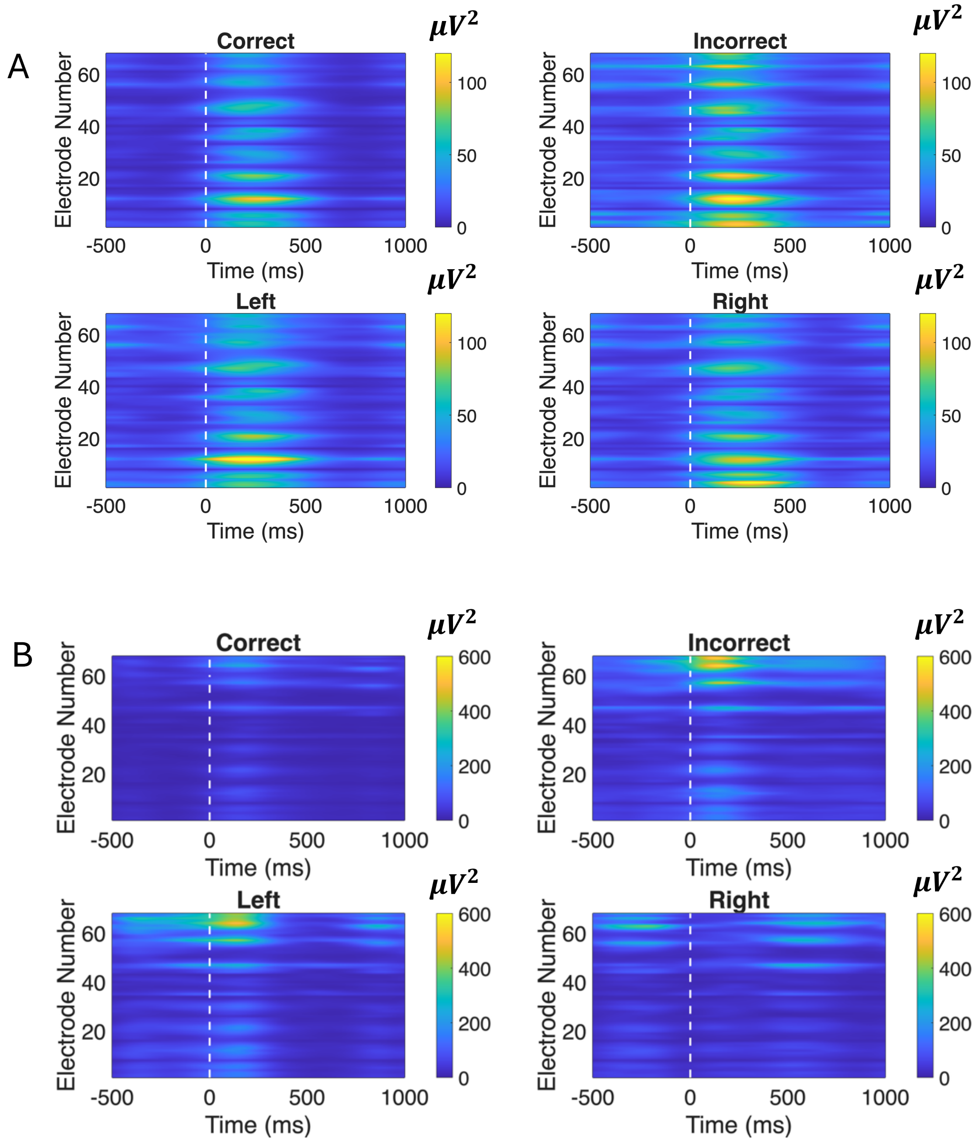

Figure S2: Raw oscillatory power of ERPs. (A) Theta band (4-7 Hz) power for all the 4 conditions: Correct, Incorrect, Left correct, Right correct; across all the electrodes (1-68); (B) Alpha band (8-12 Hz) power for all the 4 conditions: Correct, Incorrect, Left correct, Right correct; across all the electrodes (1-68).

3. Topographic plots of raw oscillatory power of ERPs across the pre-stimulus time window for all the 4 conditions for all electrodes.

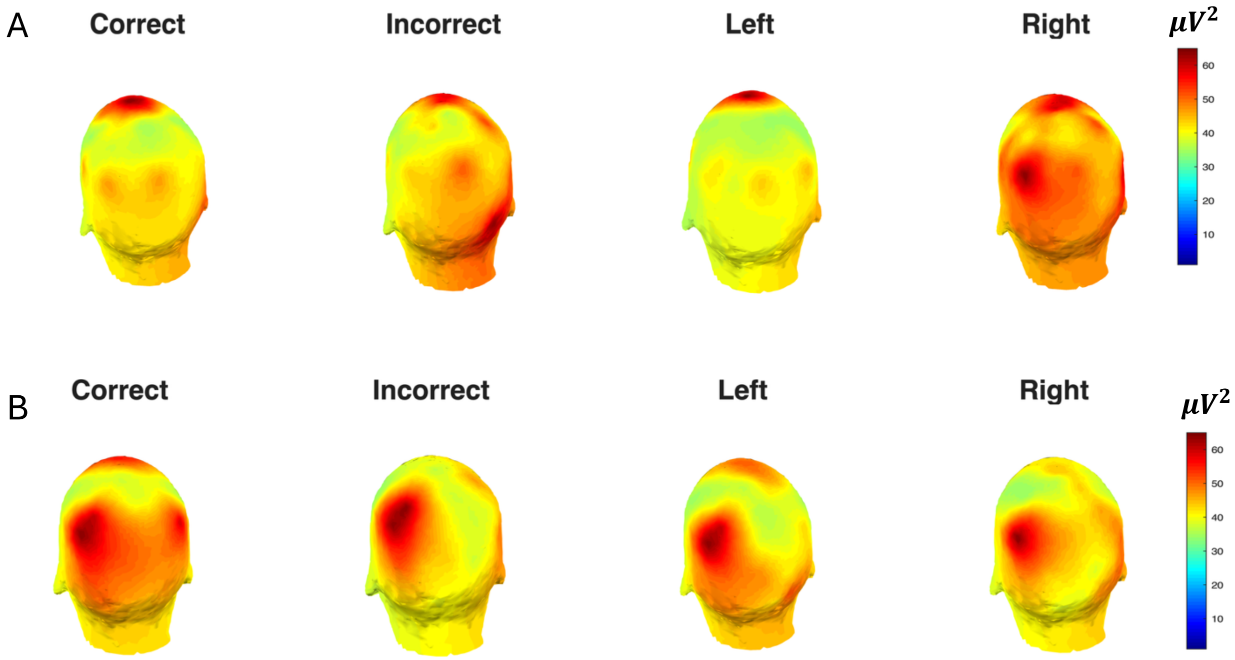

Figure S3: (A) Topographic Distribution of Raw Theta Power**.** Scalp topographies showing normalized theta band power for pre-stimulus (-500 ms to 0 ms) time window across experimental conditions. (B) Topographic Distribution of Raw Alpha Power. Scalp topographies showing normalized alpha band power for pre-stimulus (-500 ms to 0 ms) time window across experimental conditions.

4. Baseline normalized average theta power of the pre-stimulus time-period and post-stimulus time-period.

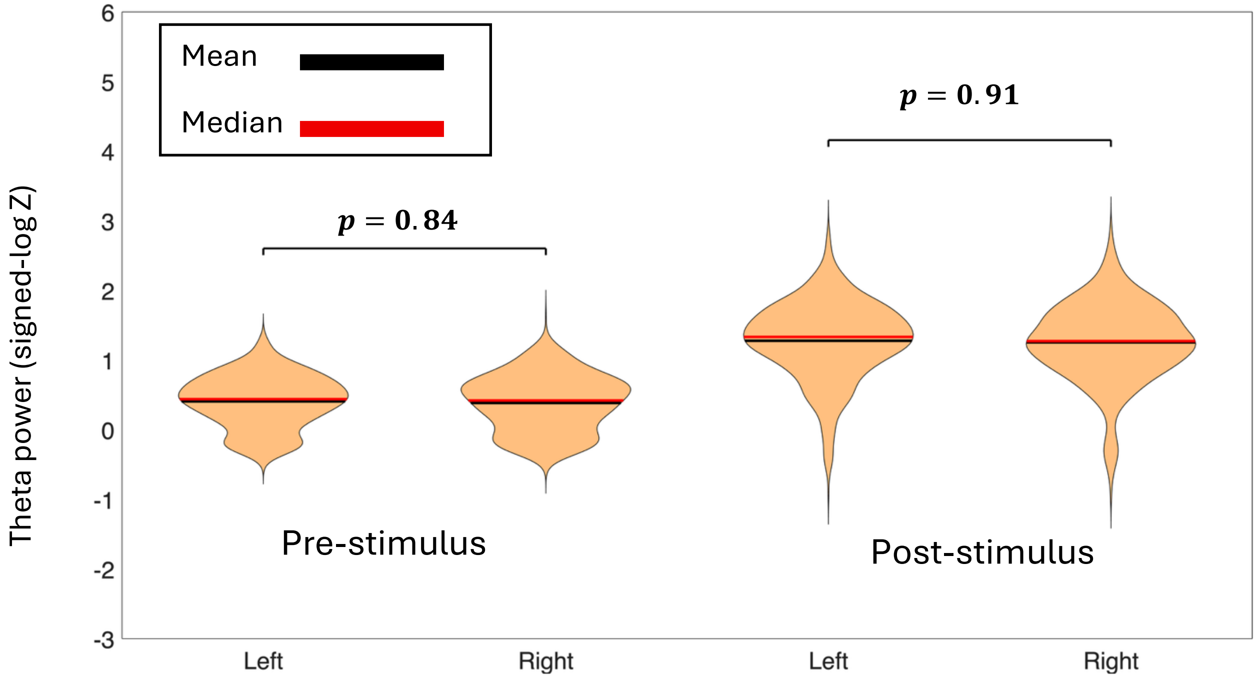

Figure S4: Baseline normalized average theta power of the pre-stimulus time-period (-500 ms to 0 ms) and post-stimulus time-period (0 ms to 500 ms) for left correct decisions vs right correct decision. The p-values represent the significance between condition pairs. The conditions are not significantly different from each other.

5. Baseline normalized average alpha power of the pre-stimulus time-period and post-stimulus time-period.

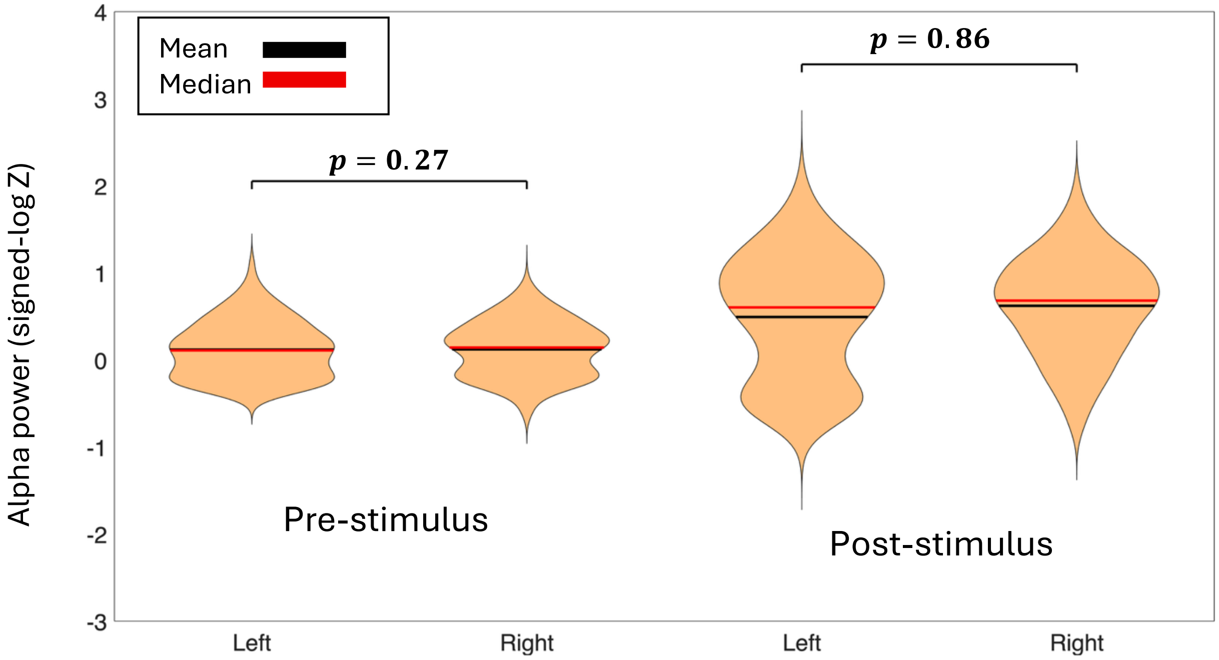

Figure S5: Baseline normalized average alpha power of the pre-stimulus time-period (-500 ms to 0 ms) and post-stimulus time-period (0 ms to 500 ms) for left correct decisions vs right correct decision. The p-values represent the significance between condition pairs. The conditions are not significantly different from each other.

6. Directional information flow between frontal and parietal cortical regions for alpha band.

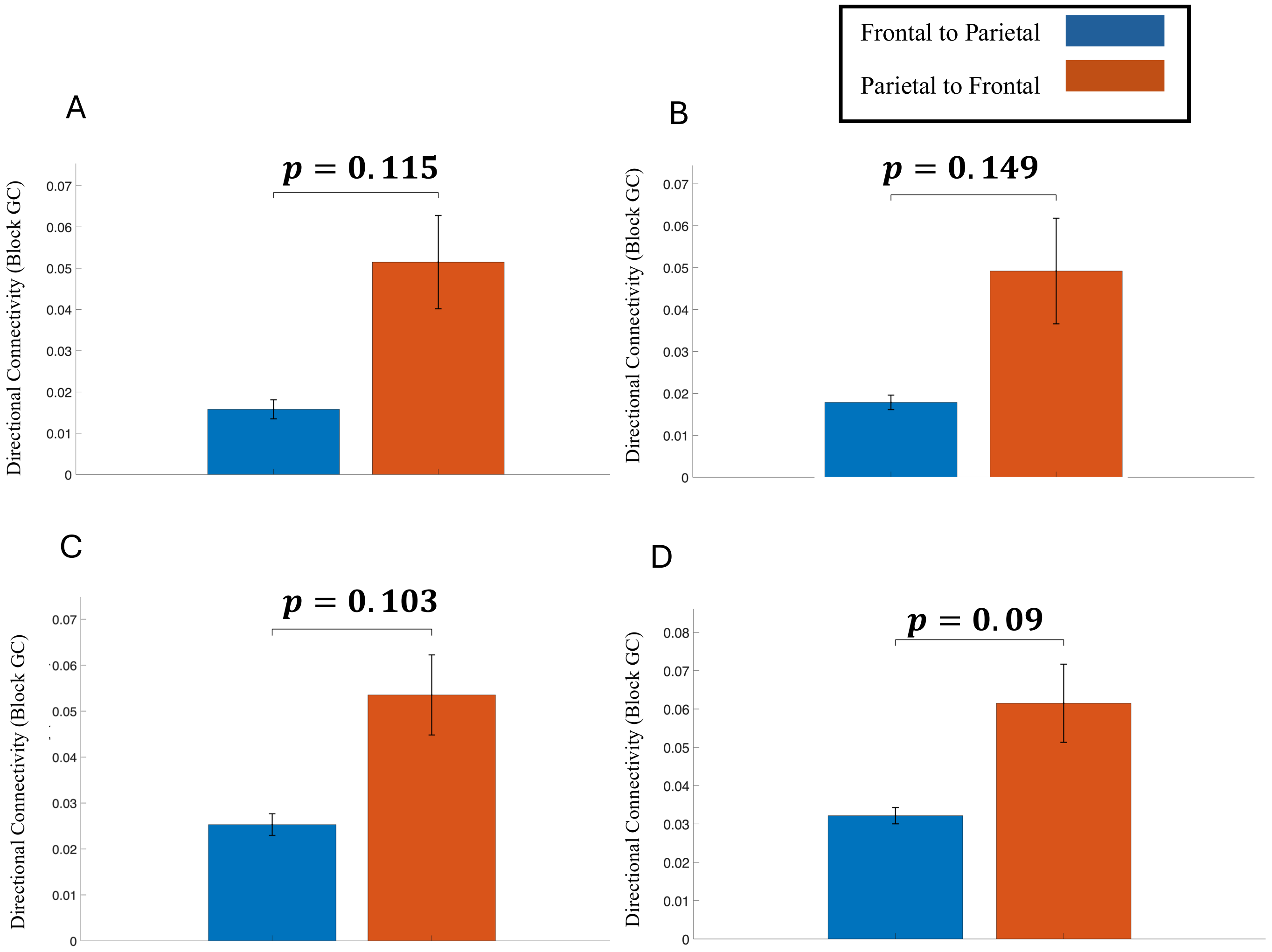

Figure S6: Directional information flow between frontal and parietal cortical regions for alpha band. (A) Correct decisions pre-stimulus period, (B) Correct decisions post-stimulus period, (C) Incorrect decisions pre-stimulus period, (D) Incorrect decisions post-stimulus period.

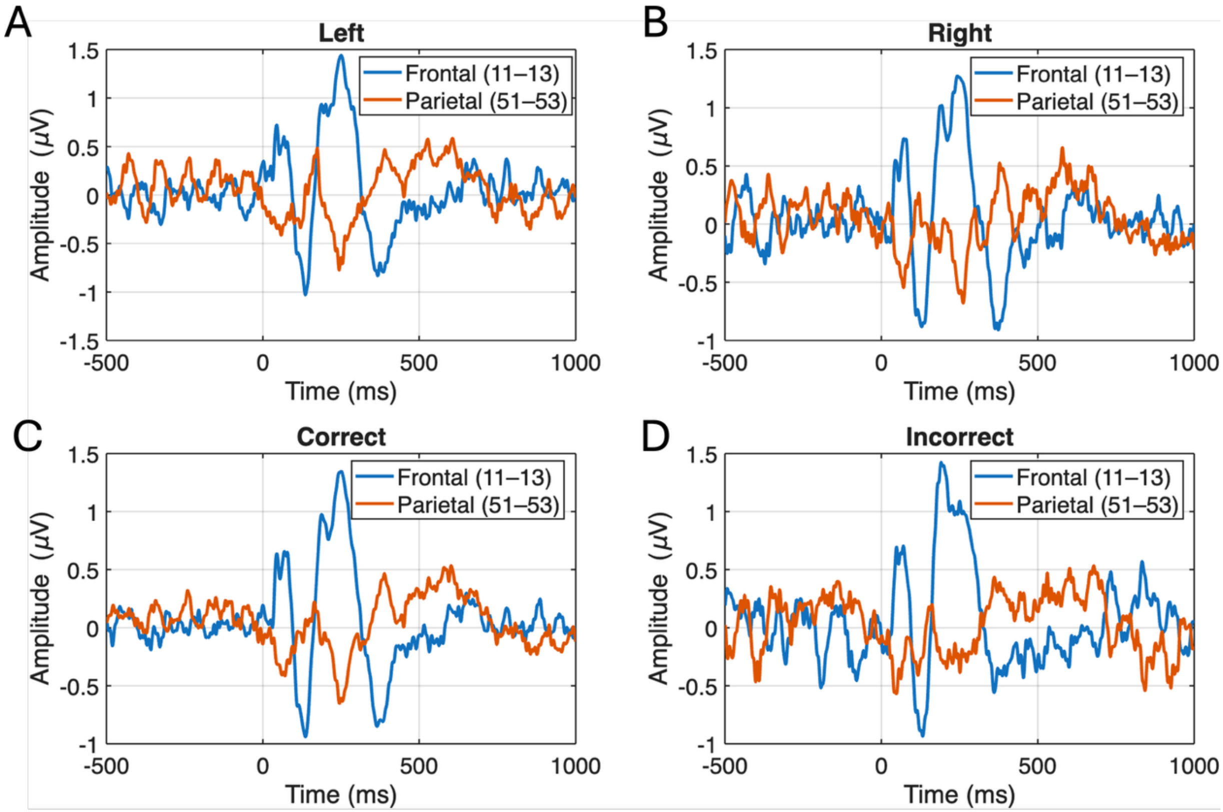

Figure S7: Grand-average event-related potentials (ERPs) across conditions and regions of interest. Grand-average ERPs are shown for the (A) Left, (B) Right, (C) Correct, and (D) Incorrect conditions. For each panel, signals were averaged across frontal electrodes (11–13; blue line) and parietal electrodes (51–53; orange line). Time is shown relative to stimulus onset.
